# Deforestation in Colombian protected areas increased during post-conflict periods

**DOI:** 10.1038/s41598-020-61861-y

**Published:** 2020-03-18

**Authors:** N. Clerici, D. Armenteras, P. Kareiva, R. Botero, J. P. Ramírez-Delgado, G. Forero-Medina, J. Ochoa, C. Pedraza, L. Schneider, C. Lora, C. Gómez, M. Linares, C. Hirashiki, D. Biggs

**Affiliations:** 1 Department of Biology, Faculty of Natural Sciences and Mathematics. Universidad del Rosario, Kr 26 No 63B-48, 111221 Bogotá, D.C. Colombia; 2 Ecología del Paisaje y Modelación de Ecosistemas-ECOLMOD, Departamento de Biología, Universidad Nacional de Colombia, Cra. 30 # 45-03 Bogotá, D.C Colombia; 3 Institute of the Environment and Sustainability, University of California, Los Angeles, CA 90024 USA; 4 Fundación para la Conservación y el Desarrollo Sostenible, Cra 70C # 50-47 Bogotá, Colombia; 5 Natural Resource and Environmental Studies Institute, University of Northern British Columbia, 3333 University Way, Prince George, V2N 4Z9 Canada; 6 Instituto de Hidrología, Meteorología y Estudios Ambientales (IDEAM), Calle 25D No. 96B-70 Bogotá, Colombia; 7 Wildlife Conservation Society – Colombia. Avenida 5 Norte, #22 N – 11 Cali, Colombia; 8 Instituto de Investigación de Recursos Biológicos Alexander von Humboldt, Bogotá D.C, Colombia, Avenida Paseo Bolivar (Circunvalar), 16-20 Bogotá, D.C Colombia; 9 Department of Geography, Livingston Campus, Rutgers, The State University of New Jersey, Piscataway, 08854 NJ USA; 10 Environmental consultant, Cll.74#11-81, Bogotá, D.C. 111221 Colombia; 11 Environmental Futures Research Institute. Griffith University, Nathan, Queensland 4111 Australia; 12 Centre for Complex Systems in Transition, School of Public Leadership, Stellenbosch University, Stellenbosch, 7600 South Africa; 13 Department of Conservation Ecology and Entomology, Stellenbosch University, Private Bag X1, Matieland, 7602 South Africa

**Keywords:** Conservation biology, Forestry

## Abstract

Protected areas (PAs) are a foundational and essential strategy for reducing biodiversity loss. However, many PAs around the world exist on paper only; thus, while logging and habitat conversion may be banned in these areas, illegal activities often continue to cause alarming habitat destruction. In such cases, the presence of armed conflict may ultimately prevent incursions to a greater extent than the absence of conflict. Although there are several reports of habitat destruction following cessation of conflict, there has never been a systematic and quantitative “before-and-after-conflict” analysis of a large sample of PAs and surrounding areas. Here we report the results of such a study in Colombia, using an open-access global forest change dataset. By analysing 39 PAs over three years before and after Colombia’s peace agreement with the Revolutionary Armed Forces of Colombia (FARC), we found a dramatic and highly significant increase in the deforestation rate for the majority of these areas and their buffer zones. We discuss the reasons behind such findings from the Colombian case, and debate some general conservation lessons applicable to other countries undergoing post-conflict transitions.

## Introduction

The growing warfare ecology literature reports both negative and positive effects of conflict for biodiversity and the natural environment^1–3^. This also applies to deforestation, which can be either increased or decreased depending on the specific complex socio-ecological dynamics linked to the conflict itself^3–5^. Increased deforestation during conflict is reported for several regions of the world^6^, including Democratic Republic of Congo (DRC) and Liberia^7^ or Myanmar and Cambodia^8^. In some cases, conflict reduces the institutional capacity to enforce laws and effectively manage the use or protection of natural resources, e.g. as reported for Kenya^9^, DRC^10^, Nepal^11^, and Colombia^5^. In other cases, the displacement of people escaping or forced to leave conflict areas, the basic mechanism for the ‘refuge effect’^12^, can prove beneficial for habitat and biodiversity protection, e.g. by limiting the pressure of resource extraction^13–15^. The demilitarized Zone between North and South Korea is a good example of such a refuge^16^. Conflict can largely disrupt economic activities^1^, such as timber logging in Nicaragua^17^, or farming, as in Sierra Leone^18^. In the Chechen wars and in the nearby Nagorno-Karabakh conflict, agricultural land was abandoned in warzones, along with reported low re-cultivation rates after the cessation of the conflict^19,20^. In other cases post-conflict development results in higher threats to forested ecosystems than conflict itself, such as in Rwanda and Liberia, where it led to increased land grabbing and logging^21,22^. In Peru, five years after armed conflict with the Shining Path ended, average forest loss increased by 58% due to government agriculture incentives and private investments^23^. Therefore, the end of a conflict is a particularly important moment for conservation^24^.

Narcotics can also be key factors linked to deforestation dynamics, especially in Central and South America^25–27^. Deforestation hotspots and protected areas often overlap with regions of drug production and trafficking^28^. For example, McSweeney *et al*.^29^ discussed how in Central America, a drug trafficking corridor, forests are often cleared to open roads and airstrips for drug transportation, to facilitate cultivation -often in conservation and indigenous areas through land grabbing and falsifying land titles-, and to expand other narco-capitalized businesses. Studies have found that illicit crops is a direct driver of deforestation^30–32^, and that policies of forced eradication can result in exacerbating the phenomenon^33,34^. In this sense, the conservation and proper governance of territories affected by narcotics cultivation is strongly linked to the efficiency of drug policies, which intensely focus on the supply-side reduction^29,35^. In addition, poverty and the lack of economic options in rural and remote areas with weak governability is also a key factor indirectly driving deforestation through illicit crops cultivation and land grabbing linked to agricultural activities^34,36^. Communities living in regions structurally characterized by lack of infrastructural development and established stable markets, often rely on forest clearing to claim land for subsistence agriculture or more profitable illicit activities. In Colombia, for example, rural settlers and small farmers, were found to be selling deforested land, in some cases opportunistically, in others under duress, to larger, well-organized agricultural producers, who in turn expect the government to adopt land tenure policies favorable to their interests^37^.

Indeed, several overlapping and interacting drivers have shaped the history of the transformation of the Colombian natural environment. Since the XVIII century about a third of the country’s forest cover shifted to multiple productive land-uses, mostly by the introduction of the cattle culture and expansion of grazing lands, urbanization and colonization of the lowlands^38^. In recent years forest loss has been driven by multiple shifting interacting forces, influenced by intraregional variations^39^. Major drivers of deforestation are the expansion of the agricultural frontier^40^ and the transformation of forest into pastures for cattle ranching^41^. Other local causes of deforestation include the creation of roads^39,42^ and human settlements^41,43^. In the last decades, illicit activities have been also part of the driving forces behind deforestation, mainly through their relation with illegal crops^44^, mining^45^ and logging^46^. In some regions, the limited access to certain areas because of the presence of different armed groups, resulted in the creation of a strong barrier that caused biodiversity protection^47^. In particular, protected areas have been major actors affected by deforestation nationally. Some of these PAs occur in areas of armed conflict and areas of intense illegal activities^37^. They have been found to successfully reduce deforestation^44^, however, as it occurs in other tropical regions, protected areas are disproportionately located in areas of low vulnerability^48^, i.e. away from roads, in soils unsuitable for agriculture, etc.

Overall, the literature on protected areas shows diverse effects on protection of forests and nature, varying largely from one geographical region to another^49^. Some evidence reports global ineffectiveness of PAs in preserving natural habitat within their boundaries, and identified widespread inadequate resourcing, in staff and budgets^50,51^. Other studies demonstrate that globally, PAs reduce the conversion of natural land cover when compared to non-protected areas^52^, and generally contribute to the conservation of local biodiversity^53^. In the case of Colombia, some literature showed that PAs and indigenous reserves can reduce deforestation, although the magnitude of this effect varies substantially depending on other governance covariates^54,55^.

Colombia endured five decades of armed conflict with the Revolutionary Armed Forces of Colombia (FARC) until a peace agreement was signed in 2016^56^. Colombia’s circumstances – containing a large network of conservation areas and having experienced a lengthy armed conflict ending in peace – provided suitable conditions for testing the hypothesis that conflict can substitute for enforcement in protected areas (PAs) in terms of forest cover, such that peace prompts an immediate increase in illegal deforestation^57^. Our analyses below focused on deforestation in and around 39 continental PAs in Colombia, representing either terrestrial National Natural Parks (NNPs) or National Natural Reserves (NNRs). We used the high-resolution Global Forest Change dataset^58^ to estimate the extent of deforestation over three years before the peace agreement (2013, 2014, and 2015) and three years after conflict (2016, 2017 and 2018). The analysis considers both the administrative limits of PAs and a 10-km buffer zone around them. These zones are critical because they limit the pressure for habitat conversion within parks and mitigate the well-known effects of ecological isolation^59,60^. The PAs analyzed in this study as such have strict legal protection, equivalent to IUCN categories I to IV. The territory included in these typologies of protected areas has not been, at the time of its initial establishment, substantially altered by human exploitation or occupation, and represents ecosystems, geomorphological landscapes and cultural manifestations of outstanding ecological, scientific, and anthropological value.

## Results

**Figure 1:**
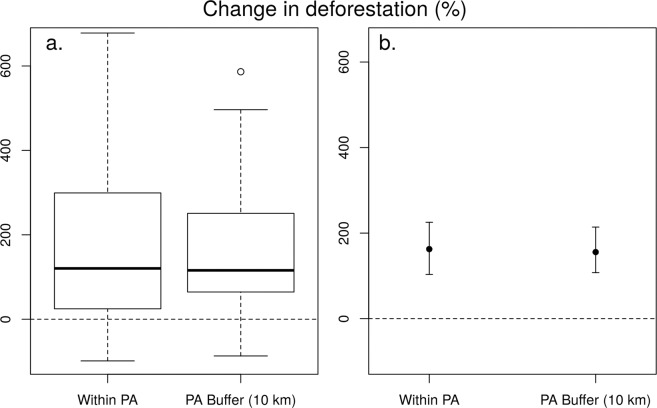
(**a**) Box-and-whisker plot of change in deforestation (%) before and after the peace agreement, for protected areas and corresponding buffer zones (10 km); n = 39. The box indicates the lower and upper quartile of data distribution; bold horizontal lines represent the median. The ‘cat’s whiskers’ (vertical dashed line) indicates the highest and lowest values of the distribution, excluding outliers (circles). (**b**) Confidence intervals (95%), based on ranks (straight vertical lines). Black circles represent the pseudomedian values from the Wilcoxon test.

**Figure 2:**
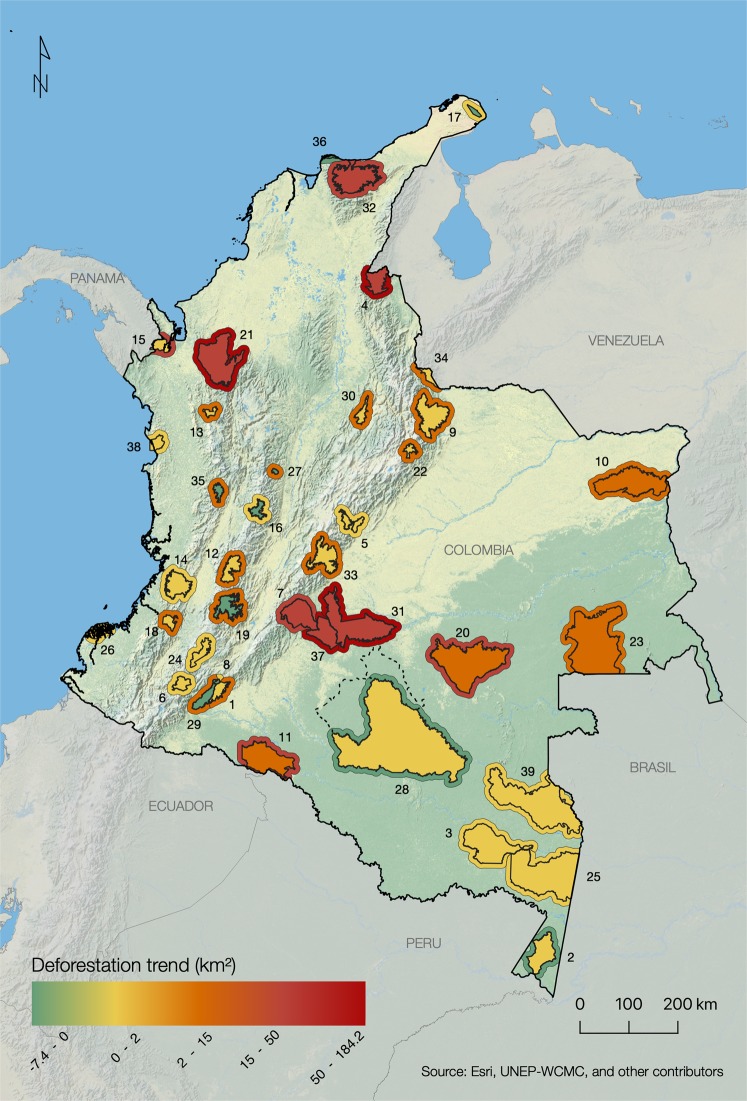
Change in deforestation extent (km^2^) before and after the peace agreement with FARC (2013–2015 vs. 2016–2018) in continental Colombian National Natural Parks and National Natural Reserves and buffer areas (10 km). Dotted line: 2018 enlargement of Serranía de Chiribiquete NNP (not used in calculations). Numbers correspond to protected area IDs, detailed in Table 1. Figure created using ArcGIS software by Esri, used herein under license.

The results are striking (Table 1; Fig. 1). Overall, in the Colombian NNPs and NNRs, 31 of the 39 PAs (79%) experienced increased deforestation in the post-conflict years (Fig. 2). This translated into a dramatic and highly significant 177% increase in the deforestation rate between the two 3-year periods (Wilcoxon V = 44, *p* = 1.81e-06), resulting in 330 km^2^ of additional loss of protected forest. In the biogeographical Amazon, of which FARC controlled vast areas, several parks suffered notably severe upswings in deforestation following the peace agreement; a prime example is the case of Serranía de la Macarena NNP (Table 1). A similar pattern was observed in the parks’ buffer areas, showing an overall post-conflict increase of +158% in forest conversion (+686 km^2^; Wilcoxon V = 37, *p* = 4.34e-07). These territories are generally represented by remote rural landscapes, where land grabbing and illicit cropping often drive land cover change^39^. Such a massive increase in natural habitat loss, often of primary forests, has potentially profound effects on biodiversity^61,62^. Additionally, at a regional level, natural habitat conversion in and around parks rapidly accelerates disruption of large ecological corridors, which act as important connectivity bridges between the biogeographical Amazon and the Andes^28^.

**Table 1:** Deforestation statistics for 39 protected areas (PAs) of Colombia (National Natural Park -NNP- or National Natural Reserve -NNR-) using Hansen *et al*. (2013) Global Forest Change dataset, *ver*. 1.6.

Name	Typology	ID	Inside the PA				Buffer of the PA (10 km)			
			Deforestation Before (km^2^)	Deforestation After (km^2^)	Deforestation Change (km^2^)	Percentage Change	Deforestation Before (km^2^)	Deforestation After (km^2^)	Deforestation Change (km^2^)	Percentage Change
			(t_0_)	(t_1_)	(t_1_ − t_0_)	(t_1_ − t_0_)/t_0_*100	(t_0_)	(t_1_)	(t_1_ − t_0_)	(t_1_ − t_0_)/t_0_ * 100
Alto Fragua Indiwasi	NNP	1	0.4	0.5	0.2	51.1%	8.0	13.5	5.5	68.7%
*Amacayacu*	NNP	2	0.8	1.7	0.9	117.9%	4.5	4.2	−0.3	−7.0%
*Cahuinari*	NNP	3	0.4	1.4	1.0	254.6%	0.7	1.2	0.5	73.0%
Catatumbo Bari	NNP	4	11.6	55.9	44.3	382.9%	42.4	131.7	89.3	210.6%
Chingaza	NNP	5	0.1	0.5	0.4	678.0%	0.5	1.1	0.6	115.8%
Complejo Volcanico Doña J. Cascabel	NNP	6	0.1	0.2	0.1	209.4%	1.7	3.4	1.7	95.9%
*Cordillera de los Picachos*	NNP	7	10.6	33.0	22.3	210.1%	9.4	55.8	46.4	496.4%
Cueva de los Guacharos	NNP	8	<0.01	0	<0.01	<0.01%	0.6	2.0	1.5	266.5%
El Cocuy	NNP	9	0.3	2.0	1.7	545.8%	1.6	6.6	5.1	323.9%
El Tuparro	NNP	10	0.6	2.8	2.2	345.0%	5.1	9.8	4.7	91.5%
*La Paya*	NNP	11	23.6	34.5	10.9	46.2%	72.2	121.0	48.8	67.7%
Las Hermosas	NNP	12	0.2	0.4	0.2	117.2%	2.1	6.7	4.7	227.2%
Las Orquideas	NNP	13	0.2	1.1	0.9	451.9%	1.9	8.8	7.0	375.8%
Los Farallones de Cali	NNP	14	0.5	1.4	0.9	170.6%	3.3	5.1	1.8	52.5%
Los Katios	NNP	15	0.4	1.3	0.9	257.5%	8.1	38.5	30.4	374.2%
Los Nevados	NNP	16	0.02	0.01	−0.01	−46.6%	3.8	4.8	1.0	27.2%
Macuira	NNP	17	0.14	0.03	−0.11	−79.3%	<0.01	<0.01	<0.01	<0.01%
Munchique	NNP	18	1.1	2.1	1.0	90.1%	9.6	23.9	14.2	147.6%
Nevado del Huila	NNP	19	0.3	0.2	−0.1	−19.1%	6.1	16.6	10.5	172.4%
*Nukak*	NNR	20	9.3	19.2	9.9	105.8%	9.3	42.0	32.7	352.9%
Paramillo	NNP	21	19.5	48.0	28.5	146.4%	26.0	78.5	52.6	202.5%
Pisba	NNP	22	0.04	0.22	0.18	459.4%	0.4	2.9	2.5	586.3%
*Puinawai*	NNR	23	6.2	11.8	5.6	89.5%	5.2	7.5	2.2	42.8%
Purace	NNP	24	0.1	0.3	0.2	170.8%	0.5	1.2	0.7	145.3%
*Rio Pure*	NNP	25	0.4	1.6	1.2	307.1%	0.4	0.8	0.4	100.3%
Sanquianga	NNP	26	0.5	0.7	0.1	25.7%	3.8	4.6	0.8	21.3%
Selva de Florencia	NNP	27	0.03	0.03	−0.01	−17.7%	2.6	9.8	7.2	279.0%
*Serrania de Chiribiquete*	NNP	28	3.6	3.8	0.2	5.1%	2.4	1.2	−1.2	−48.1%
Serrania de los Churumbelos	NNP	29	0.4	0.4	−0.1	−16.0%	12.2	22.2	10.0	81.9%
Serrania de los Yariguies	NNP	30	0.2	0.6	0.4	191.0%	2.8	15.9	13.1	468.6%
*Sierra de la Macarena*	NNP	31	41.4	91.2	49.8	120.4%	103.7	287.9	184.2	177.7%
Sierra Nevada de Santa Marta	NNP	32	7.4	30.0	22.6	304.7%	28.1	51.7	23.5	83.7%
Sumapaz	NNP	33	0.2	0.3	0.1	68.3%	1.3	3.8	2.5	198.5%
Tama	NNP	34	0.4	1.6	1.2	293.8%	3.5	7.4	3.9	108.8%
Tatama	NNP	35	0.11	0.09	−0.02	−15.8%	3.4	13.3	9.9	287.8%
Tayrona	NNP	36	1.49	0.02	−1.47	−98.7%	8.5	1.1	−7.4	−87.0%
*Tinigua*	NNP	37	37.5	159.5	122.0	325.7%	30.7	103.0	72.3	235.1%
Utria	NNP	38	0.1	0.6	0.5	341.0%	1.0	1.5	0.6	61.3%
*Yaigoje Apaporis*	NNP	39	6.7	8.2	1.6	23.6%	6.8	8.8	2.0	29.8%
**Total (39) PAs**			**186.8**	**516.9**	**330.2**	**+176.8%**	**434.2**	**1120.2**	**686.0**	**+158.0%**

Note: t_0_ = sum of deforestation extent for 2013–2015 (before peace agreement); t_1_ = sum of deforestation extent for 2016–2018 (after peace agreement). PAs names within the Colombian Amazon biogeographical region are in italics.

## Discussion

Deforestation inside Colombian PAs and in the surrounding buffer areas has accelerated with the onset of peace. Indeed, a narrative has developed about how biodiversity is threatened when peace arrives in areas previously “protected” by conflict^57^. That narrative, however, misses the point. Although conflict obstructs land development and prevents illegal usage, it cannot guarantee security for biodiversity; it ultimately is dysfunctional and emblematic of more systemic and deep-rooted problems.

**Figure 3:**
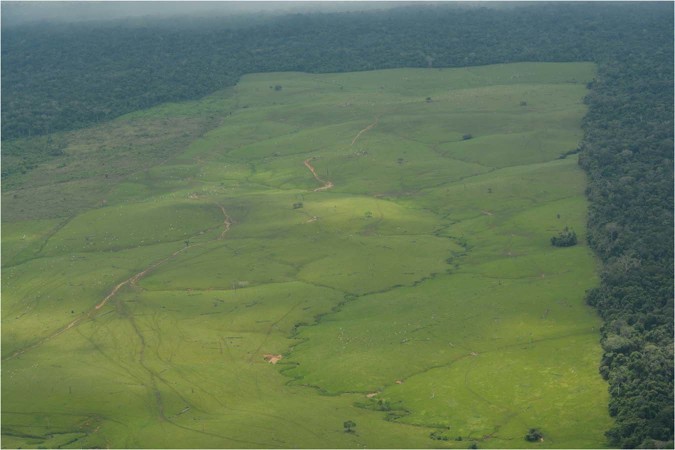
Land grabbing in southern Serranía de la Macarena NNP. Large forest patches are converted into pasture to establish livestock and claim land possession (Credits: Fundación para la Conservación y el Desarrollo Sostenible, 2017).

Several historic deforestation factors provoked this outcome, but the exit of a powerful actor that controlled a large part of the country exacerbates them. The national government’s systematic weakness in historically managing PAs and their surrounding regions owes to multiple complex and interacting causes. Crucial among these are: the country’s lack of financial, technical, and operational strength towards establishing a historical registry of illegal land occupation; its low capacity of recovering illegally grabbed land, *legally* and *physically*; and its administrative centralization that strips autonomy of regional institutions. For instance, the national government failed to ensure a functional institutional presence in several PAs. Neither the country’s law enforcement institutions (National Prosecutorial Office, police, and army) nor the Land Restitution Unit, a special administrative unit responsible for the restitution of forced dispossessed land and displaced people (Law 1448/2011), have been effective. Law enforcement agencies failed to execute the actions granted in the Colombian constitution to public conservation areas. These factors contributed to allow large-scale landowners and other illegal actors to grab land in and around PAs at low risk and establish extensive livestock systems^63^ (Fig. 3). Cattle ranching is an inexpensive method of securing possession of land by providing proof that the land is in use, which allows criminals to perform land speculation for major profits. Outside PAs, there is urgent need to define a specific policy on land property formalization for parks’ buffer areas.

**Figure 4:**
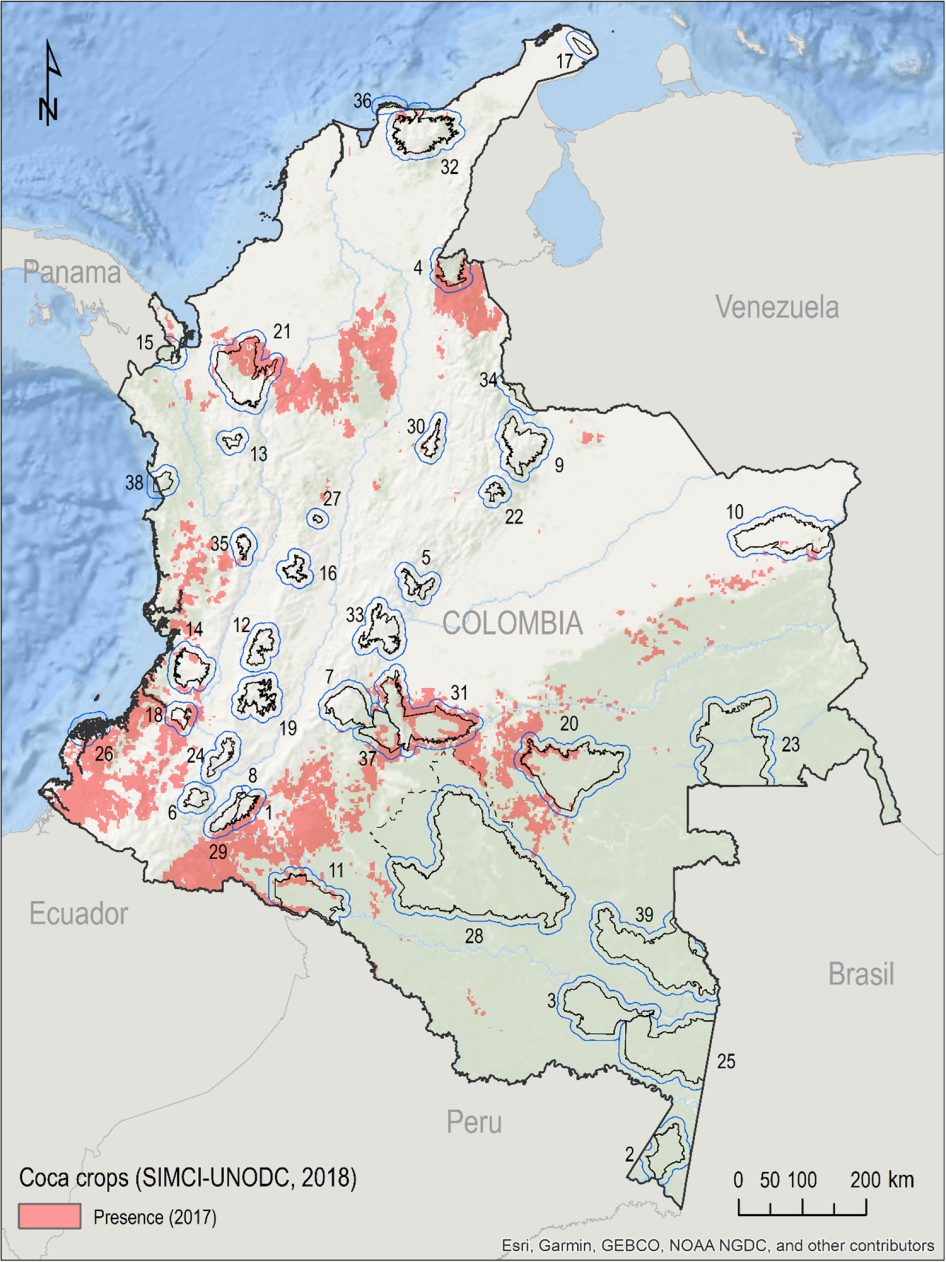
Presence of coca crops in Colombia in 2017 (source: SIMCI-UNODC, 2018). Protected area boundaries in black, buffer areas (10 km) in blue. Dotted line: 2018 enlargement of Serranía de Chiribiquete NNP (not used in calculations). Numbers correspond to protected area IDs, detailed in Table 1. Figure created using ArcGIS software by Esri, used herein under license.

The growing international demand of coca also acts as a key indirect driver of tropical deforestation^44^. Land area devoted to coca crops increased 4% from 2016 to 2017 within the Colombian system of protected areas, reaching 83 km^2^ of extension; however, there has been a relative reduction for certain PAs where authorities enlisted local communities to voluntarily substitute illicit crops with legitimate ones^44^. Fifteen of the analyzed PAs are affected by coca crops; two-thirds are concentrated in the NNPs Serranía de la Macarena (28.3 km^2^), Paramillo, and the NNR Nukak^64^ (Fig. 4). Each component of a national park’s local context, including the presence of guerrillas, paramilitary groups, and criminal bands, influences the potential for expansion of illicit crops. Furthermore, governmental limitation of the use of crop control strategies, such as fumigation, factors into the potential incentivization of illicit cropping inside the system of Colombian PAs.

Historically guerrilla groups, especially FARC, were important actors in both starting and controlling deforestation inside the system of national protected areas. From one side the guerrilla defined the areas for colonization, assigned lands among its social basis, promoted the construction of linear infrastructures and urbanizations (e.g. schools, hospitals), and greatly influenced local productive systems such as livestock and illegal cropping^65^. Currently, armed groups, especially FARC dissidents, are consolidating within new territories (e.g., in Tinigua NNP), by assigning land to farmers within and close to protected areas and promoting livestock and coca crops as an economic engine of the colonization process. These groups are re-activating old tracks used during the past conflict and opening new ones, to create a political-military transportation network. This territorial-control strategy allows consolidating a social basis for these armed groups, economic inputs for rearmament, and a population exploiting this ‘territorial security’, which also represents a source of recruitment for the guerrillas^37^. A new long-term cycle of violence is potentially incubating in these regions. At the same time, competing paramilitary groups operate with analogous behavior, establishing in areas where African oil palms (*Elaeis guineensis*) and cattle ranching are dominating, such as the low watershed of Ariari river and the towns of Puerto Lleras and San José del Guaviare (Meta department).

The current Colombian legislation regarding land planning is stratified in time and in assigning tasks and responsibilities to different institutions, while at the same time presenting often degrees of contradictions toward its objectives and plans. Especially because of its ‘strategic adaptation’ to the conflict, the Colombian government system historically presents low levels of law enforcement in rural areas^66^. This adaptation focused on social consensus towards territorial management, but under the tutelage of the armed actors of the region that were, and in many cases still are, the real owners of the decisional power within determined regions. Consequently, the system of protected areas of Colombia needs a radical transformation and development, to acquire effective enforcement of existing laws against the illegal use of natural resources and for the recovery of grabbed land. Additionally, in the current postconflict scenario, especially the ‘Victims and Lands Restitution Law’ that states that those who have been dispossessed of their lands or forced to abandon them because of the conflict have the right to their restitution, could generate a new flux of people in areas surrounding the protected areas. On the other hand, this can represent a precious opportunity for an exercise of land formalization, which would re-establish the role of the government over the illegal actors that are grabbing public land. Moreover, local population in Colombia has in general a low level of participation to decision making with regard to the use of natural resources -in the areas where it is allowed-, due to the country’s low development of participative policies and a weak capacity of financial investment and economic incentives in marginal areas.

While the specifics of Colombia may be unique, there are some general lessons that could apply to post-conflict regions characterized by poor governance. In Colombia and elsewhere following a peace settlement, it is essential that governments make a coordinated effort among all involved agencies to establish an effective physical and legal presence within and around conservation areas. Ecological restoration plans, necessary actions for degraded parks, should not just pursue strictly ecological objectives, but they should represent a clear return of the State’s institutions in the protected territories. Between PAs and unprotected land it is of primary importance to reestablish the regional ecological connectivity, at the basis of the processes that regulate at large scale the maintenance and formation of biodiversity, and that would allow Colombia to meet international commitments targets, among others, the objectives of Aichi Target 11^67^. Outside protected areas, the establishment of differential property tax regimes would reward with incentives sustainable forest use, and conversely disincentive the widely diffused unproductive cattle ranching. Degraded and low productivity areas should be at the focus of a new policy of productive restoration^68^.

The government’s presence cannot focus solely on the protection of biodiversity; it must also consider the broader social and economic needs of the local communities around PAs. Economic development is a pivotal way not only to prevent the expansion of illegal activities, but also to reduce deforestation. Government incentives that support sustainable use of forests are therefore imperative^45^. Furthermore, when security conditions permit, ecotourism presents an opportunity for local economic development within buffer zones or in PAs where legislation would allow it. Initial data already shows that in post-conflict Colombia, the number of tourists has been increasing, as well as the number of people visiting national parks^69^. This demonstrates a promising result of peace.

Exacerbated by the failure of government systems and targeted economic incentives, we face deforestation and loss of biodiversity worldwide. In Colombia, the presence of rich natural resources abused for economic growth coupled with the absence of effective governance allowed for significant recklessness. This study demonstrates an important lesson – substantial, additional support for conservation efforts and economic stabilization is imperative as regions transition from conflict to peace. When society emerges from a long period of conflict, and as economic activities resume, an uptick in deforestation is a likely and foreseeable outcome. This consequence can occur post-conflict, as in Colombia and Vietnam^70^, after the collapse of a government (e.g., the Soviet Union), or after the discovery of abundant resources in a politically weak state (e.g., oil in Nigeria). Nations undergoing such transitions require assistance to quickly re-establish protections for the natural environment. Creative bilateral agreements (e.g., debt-for-nature swaps) are a start, but aid is also needed (e.g., financial grants, technical support, policy expertise) to support governments in repatriating displaced people to their rightful lands and to restore effective rule of law within and outside conservation areas. Forests and natural systems are assets with the potential to deliver resilience and sustainable benefits to society long beyond short-term profits. Their effective conservation requires an integrative, comprehensive understanding of local communities’ needs, sustainable development, and long-term management.

## Methods

### Deforestation and protected areas data

We selected two time periods for our analysis: 2013–2015 (during conflict) and 2016–2018 (post-conflict). Deforestation data was obtained from the Global Forest Change dataset, which uses Landsat 8 OLI imagery to detect forest extent and change^58^. The raster data layer used was *Year of gross forest cover loss event (lossyear)*, which provided events of deforestation at a spatial resolution of 30 m × 30 m. All deforestation pixels referring to years not included in the analysis were excluded. Protected areas’ boundaries in geographic information systems (GIS) shapefile format were obtained from the Colombian National Natural Parks institution (http://mapas.parquesnacionales.gov.co/). Only continental forested NNPs and NNRs were selected from the database (n = 39). The 10-km buffer areas around PAs were produced in QGIS^71^ with ad-hoc routines in Python programming language to avoid buffer overlaps and double counting (Supplementary information, S1). All layers were projected to a common projection system, MAGNA-SIRGAS/Colombia Bogota zone (EPSG: 3116).

### Calculation of statistics

We used the *reduceRegion* function in Google Earth Engine^72^ to iteratively calculate the loss for each feature’s bounded area by year (Supplementary information, S2). We then calculated percentage change in deforestation areas for each PA as follows (Eq. 1):

1 $$\begin{array}{c}100\ast ({\rm{{\prime} }}{\rm{{\prime} }}{\rm{d}}{\rm{e}}{\rm{f}}{\rm{o}}{\rm{r}}{\rm{e}}{\rm{s}}{\rm{t}}{\rm{e}}{\rm{d}}\,{\rm{a}}{\rm{r}}{\rm{e}}{\rm{a}}\,{\rm{i}}{\rm{n}}\,{\rm{p}}{\rm{e}}{\rm{r}}{\rm{i}}{\rm{o}}{\rm{d}}\,{{\rm{t}}}_{1}(2016+2017+2018){\rm{{\prime} }}{\rm{{\prime} }}\\ -{\rm{{\prime} }}{\rm{{\prime} }}{\rm{d}}{\rm{e}}{\rm{f}}{\rm{o}}{\rm{r}}{\rm{e}}{\rm{s}}{\rm{t}}{\rm{e}}{\rm{d}}\,{\rm{a}}{\rm{r}}{\rm{e}}{\rm{a}}\,{\rm{i}}{\rm{n}}\,{\rm{p}}{\rm{e}}{\rm{r}}{\rm{i}}{\rm{o}}{\rm{d}}\,{{\rm{t}}}_{0}\,(2013+2014+2015){\rm{{\prime} }}{\rm{{\prime} }})\\ /{\rm{{\prime} }}{\rm{{\prime} }}{\rm{d}}{\rm{e}}{\rm{f}}{\rm{o}}{\rm{r}}{\rm{e}}{\rm{s}}{\rm{t}}{\rm{e}}{\rm{d}}\,{\rm{a}}{\rm{r}}{\rm{e}}{\rm{a}}\,{\rm{i}}{\rm{n}}\,{\rm{p}}{\rm{e}}{\rm{r}}{\rm{i}}{\rm{o}}{\rm{d}}\,{{\rm{t}}}_{0}(2013+2014+2015){\rm{{\prime} }}{\rm{{\prime} }}\end{array}$$

We performed the same analysis for the 10-km buffer zones around each PA. Graphical outputs were derived using ArcGIS 10.4.1. Boxplot (Fig. 2) and statistical tests were performed in R language^73^ using the *boxplot* and *wilcox.test* routines, respectively. Table 1 shows detailed statistics of each PA and associated buffer.

## Supplementary information

Supplementary Information.
