# Supplementary material for: Deforestation in Colombian protected areas increased during post-conflict periods
Source: Sci Rep. 2020 Mar 18;10:4971. doi: 10.1038/s41598-020-61861-y (PMC7080754; doi:10.1038/s41598-020-61861-y)
Supplement: Supplementary file 1 — Supplementary Information. [file 41598_2020_61861_MOESM1_ESM.docx]

**Deforestation in Colombian protected areas increased during post-conflict periods**

Clerici, N., Armenteras, D., Kareiva, P., Botero, R., Ramírez-Delgado, J.P., Forero-Medina, G., Ochoa, J., Pedraza, C., Schneider, L., Lora, C., Gómez, C., Linares, M., Hirashiki, C., Biggs, D.

**Supplementary Information**

**S1: code for PAs non-overlapping buffers creation**

/*SQL code for separating and attributing overlapping PAs buffers*/

/*by Marius Bottin, PhD*/

/* projecting the buffer table in WGS84*/

CREATE TABLE parks_wgs AS

SELECT gid,id_pnn,categoria,territoria,ST_transform(the_geom,4326) AS the_geom

FROM parks;

CREATE INDEX parks_wgs_idx ON parks_wgs USING GIST(the_geom);

CREATE TABLE buffers_prop_wgs AS

WITH parks_togethers AS(

SELECT ST_Union(the_geom) AS geom

FROM parks_wgs

)

SELECT p.gid,ST_Difference(ST_transform(ST_buffer(p.the_geom,10000),4326),w.geom) AS the_geom

FROM parks p,

parks_togethers w

;

CREATE INDEX buffer_prop_wgs_idx ON buffers_prop_wgs USING GIST(the_geom);

--only overlapping buffers

CREATE TABLE overlap AS

SELECT ST_Union(ST_intersection(b1.the_geom,b2.the_geom)) AS the_geom

FROM buffers_prop_wgs b1

INNER JOIN buffers_prop_wgs b2 ON ST_intersects(b1.the_geom,b2.the_geom) AND b1.gid!=b2.gid;

CREATE INDEX overlap_idx ON overlap USING GIST(the_geom);

-- Working on the raster file

CREATE TABLE overlap_sep AS

SELECT ST_intersection(b1.the_geom,b2.the_geom) AS the_geom

FROM buffers_prop_wgs b1

INNER JOIN buffers_prop_wgs b2 ON ST_intersects(b1.the_geom,b2.the_geom) AND b1.gid!=b2.gid;

CREATE INDEX overlap_sep_idx ON overlap_sep USING GIST(the_geom);

CREATE TABLE pixel_polygons AS

SELECT rid,(ST_PixelAsPolygons(ST_Clip(rast,the_geom))).*

FROM overlap o

JOIN for_cov f ON ST_intersects(rast,the_geom);

CREATE INDEX pixel_polygons_idx ON pixel_polygons USING GIST(geom);

DELETE FROM pixel_polygons p

USING overlap o

WHERE NOT ST_intersects(geom,o.the_geom);

ANALYZE pixel_polygons;

-- determining the closer park for each pixel in the overlapping buffers

SELECT AddGeometryColumn ('public','pixel_polygons','ct_geom',4326,'POINT',2);

ALTER TABLE pixel_polygons

ADD COLUMN closest int;

UPDATE pixel_polygons

SET ct_geom=ST_centroid(geom);

CREATE INDEX pixel_polygons_ct_idx ON pixel_polygons USING GIST(ct_geom);

WITH pixel_closest AS(

SELECT DISTINCT ON (rid,x,y) rid,x,y,gid

FROM pixel_polygons p

JOIN parks_wgs b ON ST_dwithin(ct_geom,the_geom,0.1)

ORDER BY rid,x,y,ST_distance(ct_geom,the_geom)

)

UPDATE pixel_polygons p

SET closest=c.gid

FROM pixel_closest c

WHERE c.rid=p.rid AND c.x=p.x AND c.y=p.y;

-- Grouping pixels

CREATE TABLE influence_on_overlap

(

gid int

);

SELECT AddGeometryColumn ('public','influence_on_overlap','ct_geom',4326,'MULTIPOLYGON',2);

INSERT INTO influence_on_overlap

SELECT closest, ST_Multi(ST_Union(ST_buffer(geom,0.000001))) AS the_geom

FROM pixel_polygons p

GROUP BY closest

;

-- final buffers

CREATE TABLE final_buffers

(

gid int PRIMARY KEY,

id_pnn text,

objectid int,

nombre text

);

SELECT AddGeometryColumn ('public','final_buffers','the_geom',4326,'MULTIPOLYGON',2);

INSERT INTO final_buffers

WITH tmp AS(

SELECT p.gid,p.id_pnn,p.objectid,p.nombre, ST_Union(ST_intersection(ST_Difference(b.the_geom,o.the_geom),b.the_geom),i.ct_geom) AS the_geom

FROM buffers_prop_wgs b

CROSS JOIN overlap o

LEFT JOIN influence_on_overlap i ON i.gid=b.gid

JOIN parks p on p.gid=b.gid

WHERE i.ct_geom IS NOT NULL

), dump AS(

SELECT gid,id_pnn,objectid,nombre,(ST_Dump(the_geom)).geom

FROM tmp

WHERE ST_geometrytype(the_geom)='ST_GeometryCollection'

)

SELECT gid,id_pnn,objectid,nombre,ST_Multi(ST_Union(geom))

FROM dump

WHERE ST_geometrytype(geom)='ST_Polygon'

GROUP BY gid,id_pnn,objectid,nombre

UNION

SELECT gid,id_pnn,objectid,nombre,ST_Multi(the_geom)

FROM tmp

WHERE ST_geometrytype(the_geom)!='ST_GeometryCollection'

;

INSERT INTO final_buffers

SELECT p.gid,p.id_pnn,p.objectid,p.nombre, ST_Multi(b.the_geom)

FROM buffers_prop_wgs b

CROSS JOIN overlap o

LEFT JOIN influence_on_overlap i ON i.gid=b.gid

JOIN parks p on p.gid=b.gid

WHERE i.ct_geom IS NULL

;

**S2: code for deforestation statistics extraction in Google Earth Engine**

// IMPORT SELECTED WPA AS A GOOGLE FUSION TABLE AND CALCULATE FOR LOSS ON GOOGLE EARTH ENGINE

// by Claire Hirashiki, modified from work of Rodrigo E. Principe

//import the selected Hansen dataset for tree cover

var gfc2018 = ee.Image('UMD/hansen/global_forest_change_2018_v1_6')

.select(['treecover2000','lossyear']);

// determine list of years for filter iteration

var years = ee.List.sequence(13, 18);

// set the scale

var scale = 30;

// import the protected areas as a feature collection

var wpa = ee.FeatureCollection('ft:1hTSHUIsDuJ1UztWfWsZIP1acleUMzBet3OsZns37');

// for buffer of 10km

// replace Feature Collection with ID: 1aKNdhQ1X7m83Kzthw9uQU3ctXmairyo9_yXhV8gf

// for total Colombia area

// replace Feature Collection with ID: 1PwPFPEnLs-xt827fYVbnNYevbSunruToRjx0hif5

// calculate the area of tree cover

var treeCover = gfc2018.select(['treecover2000']);

// tree cover layer is scale of 0-100, so divide by 100 for area in hectares

treeCover = treeCover.divide(100);

var areaCover = treeCover.multiply(ee.Image.pixelArea())

.divide(10000).select([0],["areacover"])

// create variable for total loss area

var loss = gfc2018.select(['lossyear']);

var areaLoss = loss.gt(0).multiply(ee.Image.pixelArea()).multiply(treeCover)

.divide(10000).select([0],["arealoss"]);

// create variable for the final image

var total = gfc2018.addBands(areaCover)

.addBands(areaLoss)

// create variable for coverage by feature

var wpaSums = areaCover.reduceRegions({

collection: wpa,

reducer: ee.Reducer.sum(),

scale: scale,

});

var addVar = function(feature) {

// iterate over the years

var addVarYear = function(year, feat) {

year = ee.Number(year).toInt()

feat = ee.Feature(feat)

// change year iteration number to the year itself

var realYear = ee.Number(2000).add(year)

// filter year to select and apply mask

var filtered = total.select("lossyear").eq(year)

filtered = total.updateMask(filtered)

// reduce variables

var reduceFeature = filtered.reduceRegion({

geometry: feature.geometry(),

reducer: ee.Reducer.sum(),

maxPixels: 1e13,

scale: scale,

})

// find loss and add properties

var loss = ee.Number(reduceFeature.get("arealoss"))

var name = ee.String("loss_").cat(realYear.format())

var cond = loss.gt(0)

return ee.Algorithms.If(cond,

feat.set(name, loss),

feat)

}

// iterate and create new feature

var newFeature = ee.Feature(years.iterate(addVarYear, feature))

return newFeature

}

// generate new feature collection

var wpa_colombia = wpaSums.map(addVar);

// export data as a csv to Google Drive

Export.table.toDrive({

collection: wpa_colombia,

description: 'wpa_colombia',

fileFormat: 'CSV'

});print

// Note: Google Fusion Tables have been discontinued as of December 3, 2019
